# Spatial Distribution of Soil Bacterial Communities Along an Altitudinal Gradient in Alpine Meadows of the Northeastern Qinghai–Tibet Plateau and Their Relationship with Environmental Factors

**DOI:** 10.3390/biology15060494

**Published:** 2026-03-20

**Authors:** Wenfang Chen, Huichun Xie, Shuang Ji, Yue Zhang, Xunxun Qiu, Zhiqiang Dong, Jiaxiang Xu

**Affiliations:** 1 School of Life Sciences, Qinghai Normal University, Xining 810008, China; 15110929276@163.com (W.C.);; 2 Qilian Mountain Southern Slope Forest Ecosystem Research Station, Haidong 810500, China; 3 Key Laboratory of Medicinal Animal and Plant Resources of Qinghai–Tibetan Plateau, Xining 810008, China; 4 Team of Germplasm Resources Formation Mechanism and Utilization on the Qinghai–Tibetan Plateau, Xining 810008, China; 5 Innovation and Intelligence Introduction Base for Plateau Resources Ecology and Sustainable Development, Xining 810008, China; 6 College of Geographical Sciences, Qinghai Normal University, Xining 810008, China

**Keywords:** elevational gradient, high-throughput sequencing, microbial diversity, Qinghai–Tibet Plateau, soil nutrients

## Abstract

Despite the essential role of soil microbial communities in driving nutrient cycling within alpine meadows, their distribution patterns along elevational gradients and their responses to environmental changes remain largely unexplored. To investigate this, soil samples were collected from five elevations (3300–4500 m) in the northeastern Qinghai–Tibet Plateau to analyze bacterial community composition and diversity, as well as their associations with soil physicochemical properties and enzyme activities. The results showed significant variation in bacterial community composition and diversity across elevations. *Actinomycetota*, *Pseudomonadota*, and *Acidobacteriota* were the dominant phyla at all sampling sites. Community diversity, measured by the Shannon index, generally increased with elevation, peaking at 4500 m and lowest at 3300 m. Pearson correlation analysis and redundancy analysis (RDA) indicated that soil bacterial community structure was significantly correlated with both soil nutrient factors and enzyme activities. Among these variables, total potassium, available phosphorus, catalase, and urease were strongly correlated with bacterial community differentiation. In addition, PERMANOVA results showed that elevation was the primary factor driving community variation, explaining a substantial proportion of the variation in community composition at a statistically significant level. Overall, this study highlights the distribution of bacterial communities in alpine meadow soils along an elevational gradient and their environmental associations, providing foundational data for understanding microbial community responses to environmental changes in alpine ecosystems.

## Simple Summary

Alpine meadows rely on soil bacteria that recycle plant nutrients and help maintain soil health. The study area experiences a cold and dry climate, situated on the northeastern edge of the Qinghai-Tibet Plateau. The mean annual temperature varies between −3 and 2 °C, accompanied by a brief growing season. Although soil development is slow, the accumulation of organic matter is pronounced. However, it is not well understood how these bacteria change with altitude. To investigate how environmental conditions shape microbial communities in high-mountain ecosystems, soil samples were collected from alpine meadows along an elevational gradient on the northeastern Qinghai–Tibet Plateau. The aim was to assess shifts in soil bacterial community composition with altitude and to explore how these shifts relate to soil nutrient status and enzyme activity. The results showed that both the types and diversity of soil bacteria varied across elevations, with higher elevations generally having greater bacterial diversity. Differences in bacterial communities were closely linked to soil nutrients and enzyme activity. These findings help improve our understanding of how soil life responds to environmental changes in alpine ecosystems and provide useful information for managing and protecting these sensitive environments in the future.

## 1. Introduction

The Qinghai–Tibet Plateau, the highest and largest plateau in the world, is widely known as the “Roof of the World” and the “Third Pole” and plays a critical role in regulating regional and global climate systems [1,2,3]. Alpine meadows are the dominant grassland ecosystem on the plateau, with wide distribution and a substantial proportion of the total grassland area. These ecosystems provide essential ecological functions, including maintaining biodiversity, conserving water resources, preserving soil integrity, and supporting regional ecological security [4,5]. Given their high sensitivity to environmental variation under ongoing climate change, alpine meadows have become a key research focus for understanding ecosystem dynamics on the plateau.

The distribution of soil microbial communities is jointly regulated by multiple environmental factors. Direct drivers primarily include soil organic matter, nutrient content, temperature, and enzyme activities; by regulating microbial growth, metabolism, and resource utilization efficiency, they directly determine community composition and diversity. Indirect drivers, such as elevation, vegetation type, microclimate, and topographic features, also exert significant influences, with the elevational gradient serving as a key natural gradient that integrates variations in temperature, moisture, radiation conditions, and vegetation characteristics. Compared with latitudinal gradients, environmental conditions vary more rapidly and over shorter spatial scales along altitudinal gradients, making them an ideal “natural experimental platform” for studying the ecological impacts of climate change [6,7]. Elevation directly influences soil environments by regulating temperature and moisture and indirectly shapes soil habitats through changes in vegetation types and soil physicochemical properties, thereby affecting the composition and diversity of soil microbial communities. Soil microorganisms are key drivers of material cycling and energy flow in grassland ecosystems, playing essential roles in soil organic matter decomposition, nutrient transformation, and biogeochemical cycling [8,9,10,11]. Soil bacteria, a major component of soil microbial communities, are highly responsive to environmental shifts; consequently, their community structure and diversity are frequently employed as bioindicators of environmental change [12]. Therefore, examining patterns of soil bacterial community variation along altitudinal gradients is crucial for understanding the responses of alpine meadow ecosystems to environmental conditions.

In recent years, increasing attention has been given to soil bacterial community variation along altitudinal gradients across different ecosystems; however, the reported patterns remain inconsistent. Some studies have documented a monotonic decline in soil bacterial diversity with increasing altitude [13,14], whereas others have observed unimodal patterns or no clear elevational gradients [15,16]. These inconsistencies may arise from differences in geographic regions, vegetation types, altitudinal ranges, and environmental contexts, suggesting that soil bacterial responses to altitudinal gradients vary significantly from region to region. Therefore, further investigation within a consistent regional framework is required to systematically elucidate the variation patterns of soil bacterial communities along altitudinal gradients in alpine meadows and to identify their underlying driving factors.

To investigate these dynamics, this study was conducted in a typical alpine meadow region on the northeastern Qinghai–Tibet Plateau, where sampling sites were established along an elevational gradient from 3300 to 4500 m. High-throughput sequencing was used to characterize the composition and diversity of soil bacterial communities. By integrating environmental variables, including soil physicochemical properties and enzyme activities, this study explores the key environmental factors driving altitudinal variation in soil bacterial communities. The study aims to elucidate the shifts in bacterial community composition and diversity along an elevational gradient, and to identify the key environmental drivers shaping these community variations. The findings are intended to provide a scientific basis for understanding the spatial patterns of soil bacteria in alpine meadows and their responses to environmental factors, as well as to offer insights for the ecological protection and sustainable management of plateau grassland ecosystems.

## 2. Materials and Methods

### 2.1. Study Area

**Figure 1:**
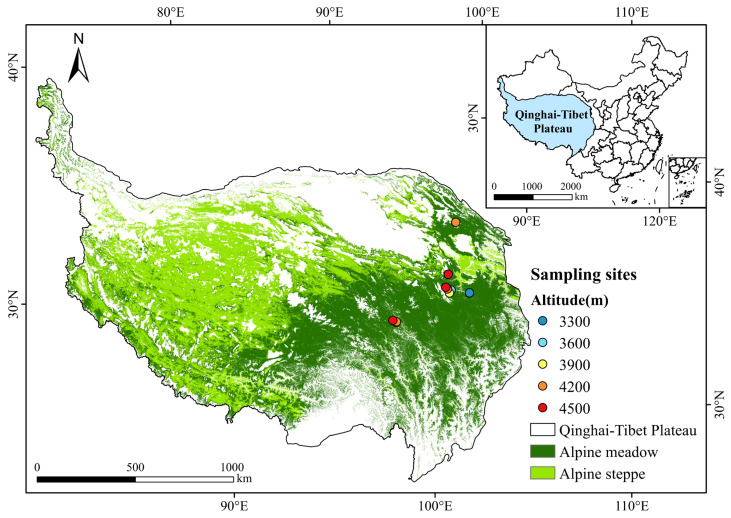
Location of the study area and distribution of sampling sites along the altitudinal gradient on the northeastern Qinghai–Tibetan Plateau.

The study area is located in the alpine meadow region of the northeastern Qinghai–Tibet Plateau and is primarily influenced by a continental plateau climate. The climate is cold and relatively arid, with a mean annual temperature ranging from approximately −3 to 2 °C and an annual precipitation of 300–500 mm, most of which occurs during the growing season (June–September). The region experiences strong solar radiation and pronounced diurnal temperature fluctuations, characteristic of alpine environments. The topography is highly undulating, with a well-defined altitudinal gradient spanning approximately 3200–5000 m. With increasing elevation, temperature decreases, the vegetation growing season shortens, and ecological factors such as soil moisture and litter input exhibit clear gradient-related changes. Vegetation across study sites is relatively consistent and dominated by alpine meadow communities. The dominant species primarily consisted of perennial herbs such as *Kobresia pygmaea*, *Kobresia humilis*, and *Carex globularis*. Within this consistent regional setting, sampling sites were established along the altitudinal gradient (Figure 1), with elevation serving as the primary environmental variable for analyzing variations in soil bacterial community composition and diversity across different elevations.

### 2.2. Soil Sample Collection and Processing

Soil samples were collected during the peak growing season of alpine meadows in August 2024. Sampling sites were established along an altitudinal gradient ranging from 3300 to 4500 m at intervals of 300 m, resulting in five elevation levels (3300, 3600, 3900, 4200, and 4500 m). Two sampling sites each were established at 3300 and 3600 m, four sites at 3900 m, and six sites at both 4200 and 4500 m (Table 1). To minimize environmental heterogeneity, sampling sites were selected to have similar grassland utilization patterns, minimal human disturbance, and comparable slope and aspect. At each site, three replicate sampling areas were randomly selected. Within each sampling area, three 1 m × 1 m quadrats were established to survey dominant plant species. Soil samples were collected from the 0–20 cm topsoil layer using a 5 cm diameter stainless steel auger following an “S”-shaped random sampling pattern. Within each sampling plot, all collected soil cores were thoroughly mixed to form a single composite sample. A total of 60 composite soil samples were obtained across five elevational gradients for subsequent analyses. The samples were placed into sealed bags and transported to the laboratory. In the laboratory, each sample was passed through a 2 mm sieve to remove plant debris and gravel, and then homogenized thoroughly. The prepared soil was divided into two subsamples: one subsample was transferred into 10 mL sterile centrifuge tubes and stored at −80 °C for analysis of soil bacterial diversity and community structure, while the other subsample was air-dried for determination of soil physicochemical properties.

### 2.3. Analytical Methods

#### 2.3.1. Soil Physicochemical Properties and Enzyme Activities

Soil physicochemical properties were determined following the methods described by Zhang et al. [17] and Bao [18]. We measured total nitrogen (TN) using a flow analyzer after digestion with sulfuric acid and an accelerator. Total phosphorus (TP) was quantified via molybdenum antimony colorimetry following digestion with sulfuric and perchloric acids. Total potassium (TK) was measured using the sodium hydroxide fusion method. Alkali-hydrolyzable nitrogen (AN) was determined by the alkaline hydrolysis diffusion method. Available phosphorus (AP) was extracted with sodium bicarbonate and then measured by the molybdenum antimony anti-colorimetric method. Available potassium (AK) was extracted with ammonium acetate and determined by atomic absorption spectrophotometry. Soil enzyme activities were assayed following the protocols described by Guan [19] and Lu [20]. All assays were performed with three replicates, and treatments without soil were used as blank controls. Urease (URE) activity was determined using the indophenol blue colorimetric method. Invertase (INV) activity was measured by the 3,5-dinitrosalicylic acid colorimetric method. Catalase (CAT) activity was determined using the potassium permanganate titration method. Alkaline phosphatase (AKP) activity was assayed using the disodium phenyl phosphate colorimetric method. All measurements were conducted in accordance with the respective standard methods

#### 2.3.2. Microbial DNA Extraction and High-Throughput Sequencing

Soil bacterial communities were characterized via high-throughput sequencing conducted by Shanghai Meiji Biotechnology Co., Ltd. (Shanghai, China). Total genomic DNA was extracted from soil samples using the E.Z.N.A.^®^ Soil DNA Kit (Omega Bio-tek, Norcross, GA, USA) according to the manufacturer’s protocol, with DNA integrity, concentration, and purity verified by 1% agarose gel electrophoresis and spectrophotometric analysis. The V3–V4 hypervariable region of the 16S rRNA gene was amplified using barcoded primers 338F (5′-ACTCCTACGGGAGGCAGCAG-3′) and 806R (5′-GGACTACHVGGGTWTCTAAT-3′) [21]. Following purification and quantification, amplicons were used to construct sequencing libraries with the NEXTFLEX Rapid DNA-Seq Kit and sequenced on the Illumina NextSeq 2000 platform (Illumina, Inc., San Diego, CA, USA). Bioinformatic processing included quality filtering of paired-end reads with fastp [22], assembly with FLASH [23], and clustering into operational taxonomic units (OTUs) at 97% similarity using UPARSE v7.1 [24,25]. Prior to clustering, singleton OTUs were removed to minimize the impact of sequencing errors, and chimeric sequences were detected and eliminated using the software’s de novo algorithm. To maintain comparability with historical datasets from analogous extreme environments, we employed the conventional 97% OTU clustering strategy rather than single-nucleotide resolution ASV methods (e.g., DADA2). Given that microorganisms in the studied extreme environments may harbor multiple 16S rRNA gene copies with subtle sequence variations, the 97% OTU approach consolidates these microheterogeneous sequences from the same organism into functional units, thereby preventing overestimation of diversity at the ASV level that could arise from intragenomic copy variations. Taxonomic assignment was performed against the SILVA 16S rRNA database (v138.2) using the RDP classifier [26] at an 80% confidence threshold. Community composition was assessed at various taxonomic levels, and functional profiling of bacterial communities was predicted using PICRUSt2 [27] (version 2.2.0).

### 2.4. Statistical Analysis

All statistical analyses were performed using IBM SPSS Statistics 27.0 (IBM, Armonk, NY, USA) and the R (version 4.5.1) programming environment, and figures were generated using Origin 2026 and R (version 4.5.1). Prior to analysis, data normality and homogeneity of variance were assessed using the Shapiro–Wilk and Levene’s tests, respectively. For data meeting these assumptions, one-way analysis of variance (ANOVA) was used to test differences among elevational gradients, followed by Duncan’s post hoc test for multiple comparisons. Non-parametric tests were applied when normality assumptions were not met. Soil microbial α-diversity indices, including Shannon and Chao1, were calculated using the vegan package in R (version 4.5.1), and variation in soil enzyme activities and microbial α-diversity across elevational gradients was visualized using Origin 2026. Differences in microbial community structure among samples were examined using non-metric multidimensional scaling based on Bray–Curtis distance matrices, and the significance of these differences was tested using permutational multivariate analysis of variance. Differentially abundant microbial taxa across elevational gradients were identified using linear discriminant analysis effect size (LEfSe), with thresholds of LDA score > 2 and *p* < 0.05 [28], focusing on significant taxa at the phylum level. Functional pathway prediction was conducted using PICRUSt, and Kyoto Encyclopedia of Genes and Genomes (KEGG) metabolic pathway annotations were subjected to inter-group difference testing in R. Relationships between soil bacterial community structure and environmental factors were assessed using distance-based redundancy analysis (db-RDA) based on Bray–Curtis distances, and the significance of environmental factors was evaluated through permutation tests.

## 3. Results

### 3.1. Variation in Soil Physicochemical Properties Along the Elevational Gradient

Soil physicochemical properties responded differently to the altitudinal gradient (Table 2). The contents of soil TN, TP, TK, and AN did not differ significantly among elevations (*p* > 0.05), remaining relatively stable across the gradient. In contrast, soil available nutrients exhibited significant elevational variation. Both soil AP and AK differed significantly among elevations (*p* < 0.05). Specifically, AP content increased initially and then decreased with increasing elevation, reaching its peak at 4500 m, while the available potassium content exhibited a unimodal pattern along the elevational gradient, increasing to a peak at 3600 m and then declining, with the value at 3600 m being significantly higher than those at other elevations.

### 3.2. Soil Enzyme Activities Across Different Elevations

**Figure 2:**
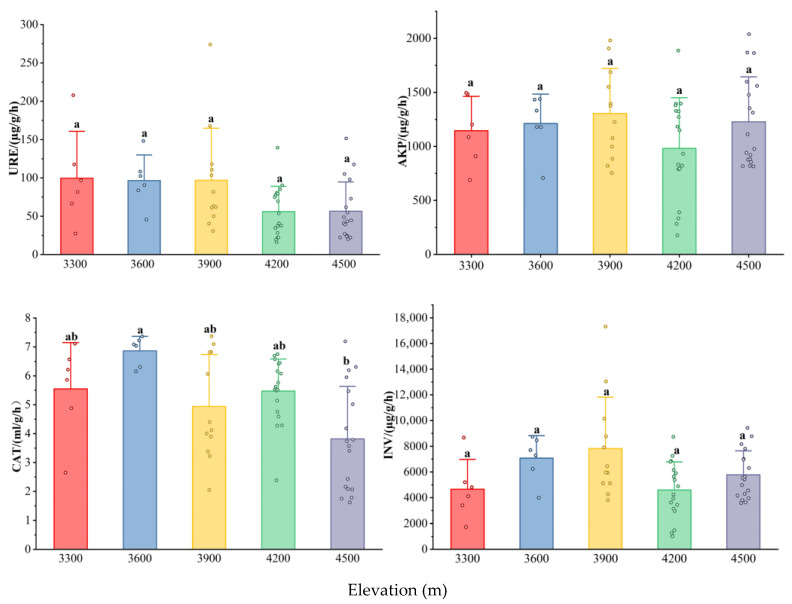
Soil enzyme activities at different elevations. Note: Bars represent the mean value, and dots represent individual data points, different lowercase letters indicate significant differences at different altitude gradients (*p* < 0.05).

Soil enzyme activities also showed distinct patterns along the elevational gradient (Figure 2) One-way ANOVA showed that soil enzyme activities responded differently to elevation (Figure 2) Urease (URE) activity, which is involved in nitrogen cycling, peaked at 3900 m, followed by a significant decline at 4200 m, and then increased again at 4500 m. Similarly, the activities of alkaline phosphatase (AKP) and invertase (INV) enzymes associated with phosphorus and carbon cycling, respectively, exhibited a peak at 3900 m, decreased significantly at 4200 m, and subsequently rose again at 4500 m. As for catalase (CAT) activity, it showed a fluctuating trend along the elevational gradient, reaching a maximum at 3600 m, decreasing significantly at 3900 m, increasing again at 4200 m, and finally declining at 4500 m.

### 3.3. Variation in Soil Bacterial Community Composition with Elevation

**Figure 3:**
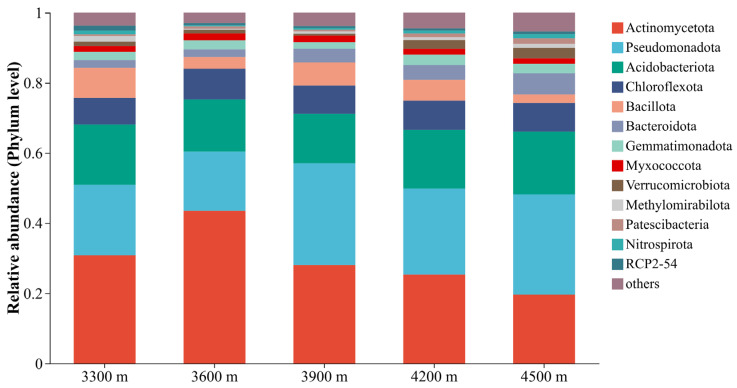
Phylum-level relative abundance of soil bacterial communities across different elevations.

A total of 3,061,088 valid sequences were obtained through high-throughput sequencing, representing 50 bacterial phyla. As shown in Figure 3, soil bacterial community composition at the phylum level varied across elevation sites. Fourteen bacterial phyla dominated the communities across all elevations. *Actinomycetota* was the most abundant phylum at all sites, maintaining consistently high relative abundance across the elevation gradient, followed by *Pseudomonadota*, *Acidobacteriota*, and *Chloroflexota*. Although these dominant phyla were present at all elevations, their relative abundances varied significantly along the elevational gradient. Specifically, *Actinomycetota* showed an initial increase with elevation, peaking at 3600 m, whereas *Pseudomonadota* fluctuated across elevations and reached its lowest relative abundance at 3600 m. Conversely, *Acidobacteriota* initially decreased and then increased with elevation, attaining its highest abundance at 4500 m. Other bacterial phyla, such as *Bacillota*, *Bacteroidota*, and *Gemmatimonadota*, occurred at relatively low abundances across all elevation sites and did not exhibit clear or consistent elevational patterns.

**Figure 4:**
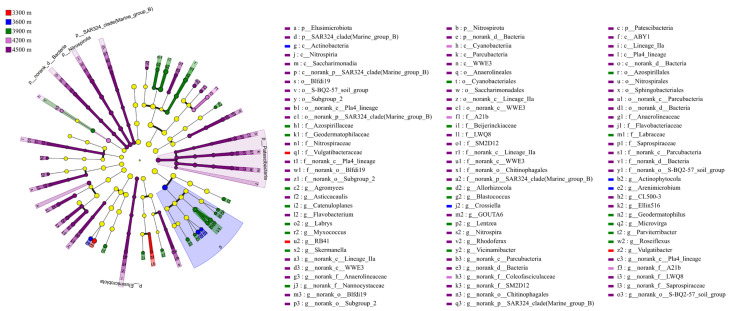
Linear discriminant analysis effect size (LEfSe) analysis of soil bacterial communities along the elevational gradient.

The LEfSe analysis revealed significant differences in soil bacterial community composition among sampling sites at different elevations (Figure 4). Using the screening criteria of LDA > 2 and *p* < 0.05, multiple indicator taxa showing differential abundance along the elevational gradient were identified across several taxonomic levels, including class, order, family, and genus. The high-elevation site (4500 m) exhibited the greatest number of differentially enriched taxa. At the class level, Elusimicrobiota, Patescibacteria, Nitrospirota, SAR324_clade (Marine_group_B), and norank_d__Bacteria showed strong discriminant effects. At the family level, Anaerolineales, Nitrospirales, Blfdi19, and S-BQ2-57_soil_group were significantly enriched. At the genus level, *Anaerolineaceae*, *Flavobacteriaceae*, *SM2D12*, and *norank_c__Lineage_IIa* were identified as representative indicator taxa at this elevation. At the mid-elevation sites (3900–4200 m), several characteristic taxa were also detected. At 3900 m, Azospirillales and Cyanobacteriales were the primary indicator taxa at the order level, while Beijerinckiaceae, Geodermatophilaceae, Labraceae, and Azospirillaceae were relatively enriched at the family level. At 4200 m, A21b emerged as the main differential taxon. In contrast, the low-elevation site (3300 m) exhibited fewer differentially abundant taxa, with *Vulgatibacteraceae* identified as a discriminant indicator taxon only at the genus level.

### 3.4. Analysis of Soil Bacterial Diversity at Different Elevations

#### 3.4.1. α-Diversity

**Figure 5:**
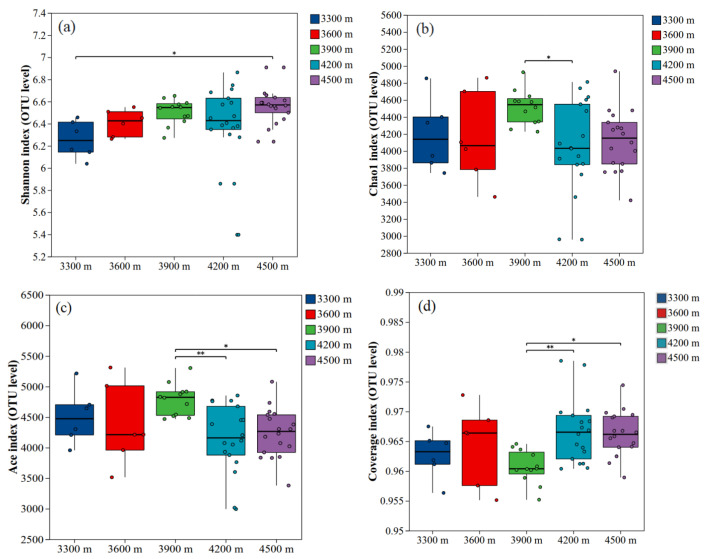
Richness and α-diversity indices of bacterial soil communities at different elevations, Bacterial Shannon index (**a**), Chao1 index (**b**), ACE index (**c**), and coverage index (**d**). Note: * Indicates significant correlation (*p* < 0.05) and ** indicates highly significant correlation (*p* < 0.01).

The Kruskal–Wallis H test was used to compare soil bacterial α-diversity indices [abundance-based coverage estimator (ACE), Chao1, coverage, and Shannon] across different elevations (3300, 3600, 3900, 4200, and 4500 m). Significant differences were observed in several α-diversity indices among elevations (Figure 5). The Shannon index (Figure 5a) varied significantly with elevation (*p* < 0.05) and showed an overall increasing trend, indicating higher bacterial diversity and at higher elevations. The ACE index (Figure 5c) also differed significantly among elevations, with values at 3900 m significantly higher than those at 4200 m, suggesting relatively greater species richness at this elevation. The Chao1 index (Figure 5b) exhibited a pattern similar to that of the ACE index, further supporting a richness peak at 3900 m. Although the coverage index (Figure 5d) varied significantly across elevations, its values were consistently close to 1, indicating sufficient sequencing depth to adequately capture soil bacterial diversity at all sites.

#### 3.4.2. β-Diversity

**Figure 6:**
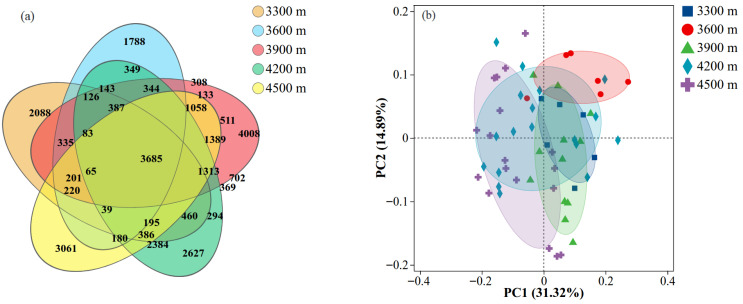
Venn diagrams (**a**) and Principal coordinate analysis (PCoA). (**b**) β-diversity patterns of soil bacterial communities at different elevations.

The Venn diagram analysis revealed a total of 209 213 were detected across soil bacterial communities at different elevations, of which 3658 OTUs were shared among all sites, accounting for 12.60% of the total (Figure 6a). Each elevation site also contained a distinct set of unique OTUs. The highest number of unique OTUs occurred at 3900 m, followed by the 4500 m and 4200 m sites, whereas the 3300 m and 3600 m sites had relatively fewer unique OTUs, indicating clear differences in community composition among elevations. Principal Coordinate Analysis (PCoA) based on Bray–Curtis distances further revealed clear separation of soil bacterial communities across elevation levels (Figure 6b). The first two principal coordinates (PCoA1 and PCoA2) explained 31.32% and 14.89% of the total variation, respectively. Samples from same elevations generally clustered closely in the ordination space, indicating relatively similar soil bacterial community structures within each elevation group. Notably, samples from the 3300 m and 3600 m sites showed strong intragroup clustering, indicating good reproducibility among replicates.

### 3.5. Functional Diversity of Soil Bacterial Communities Across Elevations

**Figure 7:**
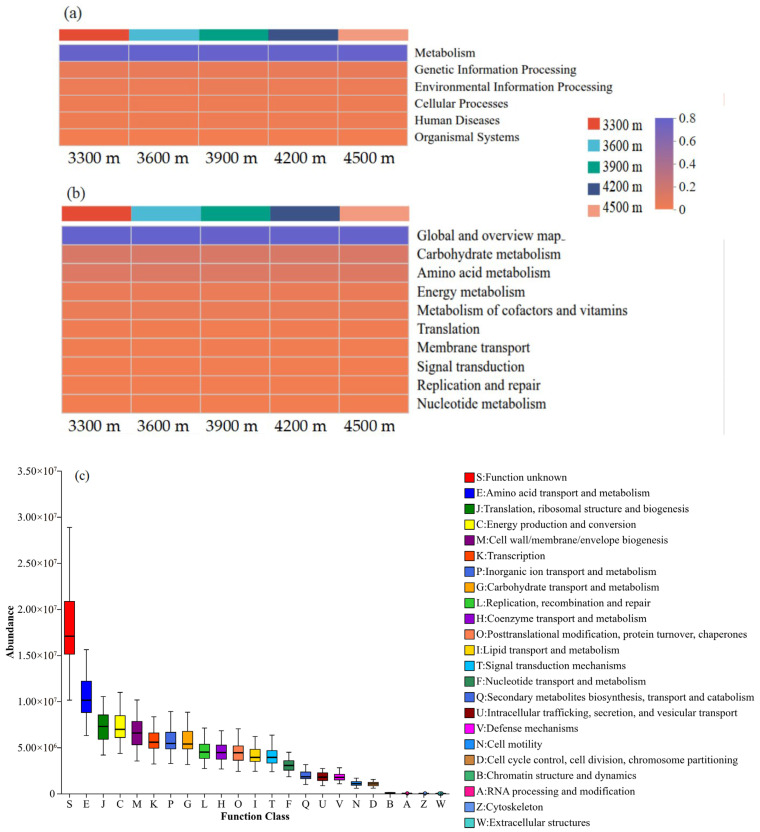
Relative abundances of predicted functional genes in soil bacterial communities across elevations: (**a**) Kyoto Encyclopedia of Genes and Genomes (KEGG) Level 1 metabolic pathways; (**b**) KEGG Level 2 metabolic pathways; (**c**) Clusters of Orthologous Groups (COG) functional categories.

Based on PICRUSt functional predictions, soil bacterial communities across different elevations were annotated to six KEGG level 1 functional categories (Figure 7a): Metabolism, Genetic Information Processing, Environmental Information Processing, Cellular Processes, Organismal Systems, and Human Diseases. Among these, Metabolism was the dominant category at all elevation sites, accounting for more than 78% of the predicted functions. At the KEGG level 2 hierarchy, 67 functional subcategories were identified (Figure 7b). Among them, “Global and overview maps” was the most abundant subcategory across all elevations, followed by Carbohydrate metabolism, Amino acid metabolism, and Energy metabolism. The relative abundances of these major functional categories varied little among elevations, indicating an overall stable functional composition. Clusters of Orthologous Groups (COG) functional annotation further classified the soil bacterial communities into 23 functional categories (Figure 7c). Excluding the “Function unknown” category (S), the dominant functional categories included Amino acid transport and metabolism (E), Energy production and conversion (C), Translation, ribosomal structure, and biogenesis (J), and Cell wall/membrane/envelope biogenesis (M), all of which showed consistently high relative abundances across elevation sites.

### 3.6. Key Factors Influencing Soil Bacterial Community Composition and Diversity

**Figure 8:**
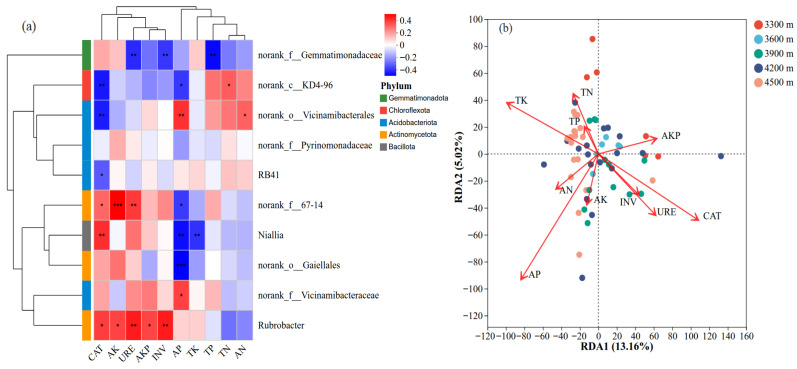
Relationships between soil bacterial community composition and environmental factors in alpine meadows across elevations: (**a**) Pearson correlation analysis; (**b**) redundancy analysis (RDA). Note: TP, total phosphorus content; URE, urease activity; INV, invertase activity; CAT, catalase activity; AP, available phosphorus content; AK, available potassium; AKP, alkaline phosphatase activity; TN, total nitrogen content; TK, total potassium content; AN, alkali-hydrolyzable nitrogen content. * Indicates significant correlation (*p* < 0.05), ** indicates highly significant correlation (*p* < 0.01), and *** indicates extremely significant correlation (*p* < 0.001).

Pearson correlation analysis was used to correlate the top 10 bacterial taxa (by relative abundance) with soil physicochemical properties and enzyme activities (Figure 8a), thereby elucidating their relationships with environmental factors. The results showed significant associations between multiple soil nutrients, enzyme activities, and bacterial taxa. Specifically, soil TN, TP, TK, AN, AP, and AK showed strong associations with the relative abundances of several bacterial taxa. CAT activity was significantly positively correlated with Niallia, Rubrobacter, and norank_f__67-14, but negatively correlated with RB41, norank_c__KD4-96, and norank_o__Vicinamibacterales. AK and URE showed significant positive correlation with Rubrobacter and norank_f__67-14, whereas URE was significantly negatively correlated with norank_f__Gemmatimonadaceae. Both phosphatase and invertase were significantly positively correlated with Rubrobacter, while invertase was negatively correlated with norank_f__Gemmatimonadaceae. In addition, AP was positively correlated with norank_o__Vicinamibacterales and norank_f__Vicinamibacteraceae, but negatively correlated with norank_c__KD4-96, Niallia, and norank_o__Gaiellales.

Redundancy analysis (RDA) further clarified the relationships between soil bacterial community structure and environmental factors (Figure 8b). The first two RDA axes explained 13.16% (RDA1) and 5.02% (RDA2) of the total community variation, yielding a cumulative explanatory power of 18.18%. The ordination results indicated that soil nutrient factors (TN, TP, TK, AN, AP, and AK) contributed substantially to the observed variation in community structure. Samples from different elevations exhibited partial separation in the RDA ordination space. Specifically, samples from 3300 m were more strongly associated with CAT, URE, and INV activities, whereas samples from 3900 m were primarily associated with INV, URE, and AP. In contrast, samples from 4500 m showed stronger associations with nutrient factors such as N, P, and K, while exhibiting relatively weaker relationships with enzyme activities.

## 4. Discussion

### 4.1. Influence of Elevation on Soil Bacterial Community Composition and Diversity

Our results show that soil bacterial communities in alpine meadows on the northeastern Qinghai–Tibet Plateau vary clearly across different elevations. Fourteen major bacterial phyla were detected across the five elevations, with *Actinomycetota*, *Pseudomonadota*, *Acidobacteriota*, and *Chloroflexota* consistently dominating. This compositional pattern is consistent with previous studies in diverse ecosystems [29,30,31], suggesting that these taxa are highly adaptable to cold alpine soil environments. The dominant bacterial groups displayed distinct responses to elevation. *Actinomycetota* increased initially and then declined at higher elevations, while *Pseudomonadota* showed fluctuating trends along the gradient. In contrast, *Acidobacteriota* exhibited higher relative abundance at higher elevations. These patterns align with previous studies indicating that *Actinomycetota* and *Pseudomonadota* thrive in environments with higher organic matter and exhibit stronger environmental adaptability, whereas *Acidobacteriota* are more prevalent under stressful conditions such as low nutrient availability and low temperatures [32]. Overall, these results highlight that bacterial taxa undergo niche differentiation along elevational gradients in alpine meadow ecosystems.

LEfSe analysis revealed bacterial taxa characteristic of specific elevational gradients. At higher elevations, groups such as Elusimicrobiota and Patescibacteria exhibited high discriminatory power. These taxa are typically adapted to oligotrophic or specialized micro-environments, indicating that high-altitude soils exert stronger environmental filtering on microbial communities. Meanwhile, the enrichment of Nitrospirota—a group involved in nitrogen cycling—at high-elevation sites further suggests the presence of niches capable of supporting their ecological functions. In contrast, mid-elevation sites were dominated by typical soil saprophytes, particularly *Actinomycetota*, whose community structure resembles that of conventional surface soils. These patterns highlight that environmental factors along the elevational gradient selectively shape bacterial communities according to their ecological preferences, providing insight into the successional mechanisms of alpine soil microbes. Variation in soil microbial diversity along elevation gradients is considered a key perspective for understanding ecosystem responses to environmental change [33]. Additionally, the α-diversity of soil bacterial communities generally increased with elevation, consistent with findings from similar alpine ecosystems [34] in. Previous studies suggest that elevational gradients modify environmental conditions such as temperature, moisture, and nutrient availability, thereby shaping patterns of soil bacterial diversity [35,36,37].

Responses of soil bacterial diversity to elevational gradients vary across different studies. For instance, Li et al. [38] reported a decline in soil bacterial diversity with increasing elevation, which they attributed to the combined stress of multiple extreme environmental factors at high elevations that may inhibit bacterial growth and community assembly. In contrast, within the elevational range examined in the present study, soil bacterial communities in alpine meadows maintained relatively high diversity, likely through species turnover and niche differentiation that facilitate adaptation to gradual environmental changes. β-diversity analyses further revealed significant differentiation in soil bacterial community structure across different elevations, indicating a strong compositional response to changes in altitude. Previous studies conducted on the Qinghai–Tibet Plateau have identified soil moisture and temperature as key drivers of microbial β-diversity [39]. Additionally, plant diversity and its spatial distribution may indirectly influence soil microbial communities through root exudates, litter inputs, and microclimatic regulation, thereby contributing to variability in microbial diversity patterns along elevational gradients.

### 4.2. Elevational Effects on the Functional Diversity of Soil Bacterial Communities

There is a close relationship between soil microbial communities and soil functionality: they not only underpin ecological processes but also respond to, and thereby reveal, alterations in functional gene composition [40]. In this study, PICRUSt was used to predict the potential functions of soil bacterial communities. At KEGG level 1, metabolic functions dominated soil bacterial communities across all elevations and consistently represented the most abundant functional category. This functional profile aligns with previous studies in alpine and grassland ecosystems [41,42], indicating that maintaining core metabolic processes is a universal characteristic of soil bacterial communities under varying environmental conditions. At the KEGG level 2, functional categories related to carbohydrate metabolism, amino acid metabolism, and energy metabolism remained highly abundant across different elevations, with only minor variation. The relative stability of this functional composition indicates that, although the taxonomic composition of the bacterial community shifted with elevation, its potential functional structure remained comparatively conserved. This pattern of “taxonomic turnover but functional conservatism” suggests that functional redundancy may play a key role in maintaining the stability of ecosystem functions in alpine meadow soils [43]. Previous studies have suggested that microbial communities can sustain essential ecosystem functions under changing environmental gradients through functional redundancy and taxa replacement [44]. In this study, shifts in soil nutrient availability (AP and TK) and enzyme activities (CAT and URE) along the elevational gradient selected for bacterial taxa adapted to distinct environmental conditions (e.g., *Actinomycetota* enriched at high elevations exhibited cold tolerance and oxidative stress resistance). However, these alternative taxa displayed substantial overlap in metabolic functions, all enriching core functional genes involved in carbohydrate and amino acid metabolism. This interpretation is further supported by COG functional annotation: the relative abundances of fundamental functional categories—including amino acid transport and metabolism (E), energy production and conversion (C), translation, ribosomal structure and biogenesis (J), and cell wall/membrane/envelope biogenesis (M)—did not differ significantly across elevations. This indicates that the functional configuration underlying basic life activities and cellular structure in soil bacterial communities is highly conserved, a finding consistent with previous studies on soil bacterial community functions in other regions [45]. It should be noted that PICRUSt predictions are based on 16S rRNA gene data and do not directly represent the actual abundance or expression of functional genes. Therefore, functional inferences should be interpreted with caution. Future studies incorporating metagenomic or metatranscriptomic approaches would provide more direct insights into the functional potential and adaptive mechanisms of soil bacterial communities in alpine environments.

### 4.3. Key Factors Influencing Soil Bacterial Communities

Soil bacterial community patterns along elevational gradients are shaped by multiple interacting environmental factors. In this study, distinct compositional and structural differences in soil bacterial communities were observed across different elevations in alpine meadows of the northeastern Qinghai–Tibet Plateau. These differences were significantly correlated with various soil nutrient factors and enzyme activity indicators, suggesting that, beyond elevation as an integrated environmental gradient, changes in soil physicochemical properties play a crucial role in driving bacterial community differentiation. In this study, AP and AK exhibited pronounced variation along the elevational gradient, consistent with previous reports from alpine regions [46]. Variation in nutrient availability may indirectly regulate bacterial community structure by influencing microbial resource acquisition strategies and niche differentiation. Previous studies indicate that mineral nutrients, particularly P and K, are key limiting factors for microbial growth and community assembly in alpine ecosystems [47,48,49]. This perspective is supported by our ordination analyses results, which showed that nutrient factors explained a significant proportion of the variation in bacterial community structure.

Soil enzyme activity is an integrated indicator of microbial metabolic potential, and its variation along elevational gradients is closely associated with bacterial community structure [50]. In this study, changes in CAT and URE activities across elevations corresponded closely with shifts in bacterial community composition, consistent with previous studies conducted along alpine elevational transects [51]. Enzymes such as URE and phosphatase are known to be highly sensitive to environmental changes, and their spatial heterogeneity often reflects microbial responses to nutrient cycling processes [52,53,54]. Our findings further suggest that soil enzyme activities, by reflecting nutrient transformation potential, are closely aligned with patterns of bacterial community differentiation along the elevational gradient.

RDA results revealed strong associations between soil bacterial community structure and multiple nutrient factors, including TN, TP, TK, AN, AP, and AK. These results indicate that soil nutrient status constitutes a key environmental background factor influencing the spatial differentiation of bacterial communities in alpine meadows. Overall, the elevational differentiation of soil bacterial communities in alpine meadows of the northeastern Qinghai–Tibet Plateau reflects the combined effects of soil nutrient availability and enzyme activity patterns.

### 4.4. Limitations of the Study

While this study revealed the distribution patterns of soil bacterial communities along an elevational gradient and their environmental associations in alpine meadows on the northeastern Qinghai-Tibet Plateau, several limitations should be acknowledged. First, the study focused solely on a single elevational gradient and did not comprehensively consider the interactive effects of factors such as vegetation type, grazing disturbance, and microclimate. As a result, the elucidation of the underlying driving mechanisms remains incomplete. Second, the sampling design was unbalanced. Due to differences in site accessibility and environmental heterogeneity, the number of sampling plots varied across elevations (*n* = 2 at 3300 m and 3600 m; *n* = 4 at 3900 m; *n* = 6 at 4200 m and 4500 m). At lower elevations (3300–3600 m), the terrain was relatively flat and vegetation homogeneous, requiring fewer plots to represent local conditions. In contrast, at mid to high elevations (3900–4500 m), increased topographic complexity and patchy vegetation necessitated a higher number of replicate plots to capture environmental variability. Although statistical methods robust to unbalanced designs (e.g., Welch’s ANOVA and db-RDA) were employed, the uneven sample sizes may still have influenced the precision of community variation estimates. Third, the coverage of environmental factors was incomplete. This study primarily analyzed soil physicochemical properties and enzyme activities, but did not delve into plant communities, root exudates, or microbial interactions. Consequently, our understanding of the environmental regulatory pathways remains limited. These limitations highlight directions for future research on soil microbial ecology in alpine meadows, with the aim of more comprehensively elucidating the distribution patterns and maintenance mechanisms of microbial communities in plateau ecosystems.

## 5. Conclusions

This study revealed a distinct ecological pattern of soil bacterial communities along an elevational gradient (3300–4500 m) in alpine meadows on the northeastern Qinghai-Tibet Plateau, characterized by increasing diversity, structural divergence, and functional stability. Although soil physicochemical properties showed no significant overall variation across the gradient, bacterial communities exhibited clear elevational differentiation: community diversity (Shannon index) increased significantly with rising elevation, indicating that high-altitude environments harbor a richer bacterial species pool. At the phylum level, *Pseudomonadota*, *Actinomycetota*, and *Acidobacteriota* were identified as the dominant taxa, with their relative abundances undergoing significant succession along the elevational gradient. Notably, PICRUSt-based functional predictions revealed that despite structural divergence, core metabolic functions (e.g., carbon, nitrogen, and energy metabolism) remained highly consistent across elevations, suggesting a potential “structurally plastic, functionally stable” adaptive strategy for microorganisms in alpine ecosystems. Redundancy analysis further identified elevation, total potassium (TK), available phosphorus (AP), catalase (CAT), and urease (URE) as key environmental factors driving community structural divergence.

In summary, this study systematically elucidates for the first time the elevational distribution patterns of soil bacterial communities in alpine meadows on the northeastern Qinghai-Tibet Plateau, revealing the sensitivity of bacterial community structure to elevation and the conservatism of their functional profiles. These findings provide a novel perspective on the ecological adaptive strategies of microbial communities in extreme environments. Furthermore, they enrich the theoretical understanding of microbial biogeography in alpine ecosystems and establish a foundational dataset for predicting the responses of soil microbial communities to climate change.

## Figures and Tables

**Table 1:** Basic topographic and vegetation characteristics of the study sites.

Plot ID	Elevation (m)	Number of Sampling Sites	Vegetation Type (Dominant Species)
A1	3300	2	*Kobresia pusilla*, *Stellera chamaejasme*, *Gentiana straminea*, *Bistorta officinalis*
		
A2	3600	2	*Kobresia pusilla*, *Anaphalis lactea*, *Bistorta officinalis*, *Oxytropis ochrocephala*
		
A3	3900	4	*Lomatogonium carinthiacum*, *Aster souliei*, *Gentiana aristata*, *Potentilla anserina*
		
A4	4200	6	*Anaphalis lactea*, *Gentiana straminea*, *Oxytropis ochrocephala*, *Elymus nutans*
		
A5	4500	6	*Anaphalis lactea*, *Astragalus strictus*, *Kobresia pusilla*, *Arenaria serpyllifolia*
		

**Table 2:** Variation in soil physicochemical properties along the altitudinal gradient.

Altitude (m)	TN (g/kg)	TP (g/kg)	TK (g/kg)	AN (mg/kg)	AP (mg/kg)	AK (mg/kg)
3300	5.86 ± 0.75 a	0.68 ± 0.02 a	17.04 ± 0.59 a	314.19 ± 43.49 a	2.79 ± 0.17 b	129.33 ± 0.17 b
3600	6.23 ± 0.30 a	0.66 ± 0.02 a	16.66 ± 0.19 a	318.99 ± 9.59 a	3.97 ± 0.48 ab	303.75 ± 0.48 a
3900	5.73 ± 0.57 a	0.72 ± 0.06 a	16.83 ± 0.36 a	344.13 ± 43.80 a	5.16 ± 0.42 a	197.75 ± 0.42 ab
4200	4.61 ± 0.55 a	0.62 ± 0.05 a	17.70 ± 0.43 a	291.06 ± 33.69 a	4.12 ± 0.33 ab	192.84 ± 0.33 ab
4500	6.83 ± 0.76 a	0.69 ± 0.04 a	17.63 ± 0.54 a	466.90 ± 65.94 a	5.92 ± 0.50 a	163.42 ± 0.51 b

Note: Different lowercase letters within the same column indicate significant differences among elevational gradients (*p* < 0.05).

## Data Availability

The original contributions presented in this study are included in the article. Further inquiries can be directed to the corresponding authors.

## Abbreviations

The following abbreviations are used in this manuscript:

ACE	Abundance-based coverage estimator
AK	Available potassium
AKP	Phosphatase
AN	Alkali-hydrolysable nitrogen
ANOVA	Analysis of variance
AP	Available phosphorus
db-RDA	Distance-based redundancy analysis
CAT	Catalase
COG	Clusters of Orthologous Groups
INV	Invertase
KEGG	Kyoto Encyclopedia of Genes and Genomes
LEfSe	Linear discriminant analysis effect size
OTU	Operational taxonomic unit
PCoA	Principal coordinate analysis
PCR	Polymerase chain reaction
RDA	Redundancy analysis
TK	Total potassium
TN	Total nitrogen
TP	Total phosphorus
URE	Urease
